# Crosslinking Dynamics and Gelation Characteristics of Photo- and Thermally Polymerized Poly(Ethylene Glycol) Hydrogels

**DOI:** 10.3390/ma13153277

**Published:** 2020-07-23

**Authors:** Jungmoon Sung, Dong Geun Lee, Sukchin Lee, Junyoung Park, Hyun Wook Jung

**Affiliations:** 1 Department of Chemical and Biological Engineering, Korea University, Seoul 02841, Korea; jmsung@sk.com (J.S.); leedg@grtrkr.korea.ac.kr (D.G.L.); jypark@grtrkr.korea.ac.kr (J.P.); 2 Analysis Platform, R&D Center, SK Innovation, Daejeon 34124, Korea; hoopoe@sk.com

**Keywords:** PEG hydrogel, UV and thermal crosslinking, real-time FT-IR, rheological properties, gelation properties

## Abstract

The crosslinking behaviors and gelation features of poly(ethylene glycol) (PEG) hydrogels were scrutinized during the UV and thermal polymerizations of mixtures of poly(ethylene glycol) methacrylate (PEGMA, monomer) and poly(ethylene glycol) dimethacrylates (PEGDMAs, crosslinkers). The real-time crosslinking behavior of the PEG hydrogels was quantified as a function of the UV irradiation time and reaction temperature during the UV and thermal polymerization, respectively, using real-time FT-IR spectrometry and rotational rheometry. The gelation characteristics of UV- and thermally crosslinked hydrogels were compared through the analysis of the gel fraction, swelling ratio, surface hardness, and the loading and release of rhodamine-B. The gelation properties of the cured hydrogel films were suitably correlated with the real-time rheological properties and crosslinked network state of the PEG mixtures. The crosslinking and gelation properties of the cured hydrogels could be optimally tuned by not only the molecular weight of the crosslinker but also the UV or thermal polymerization conditions.

## 1. Introduction

Hydrogels are widely applied in antifouling and biomedical applications, such as super-absorbents, sensing, drug delivery, fluid control, artificial muscles, nerve regeneration, and tissue engineering [1,2,3]. In particular, many studies related to hydrogels have focused on drug delivery systems, which commonly employ poly(propylene oxide), poly(*N*-isopropylacrylamide), poly(lactide-co-glycolic acid), poly(propylene fumarate), polycaprolactone, or block-copolymer combinations [4]. Recently, poly(ethylene glycol) (PEG) hydrogels have attracted attention because of their advantageous features, such as non-toxicity, good water solubility, biocompatibility, and highly tunable properties; such hydrogels have been applied in drug delivery, wound healing, and various biomedical applications [5,6,7]. In addition, the crosslinking density of PEG hydrogels can be favorably tuned by changing the monomer (e.g., poly(ethylene glycol) methacrylate (PEGMA), poly(ethylene glycol) acrylic amide, vinyl alcohol, methyl methacrylate, and methacrylic acid) and/or the crosslinker (e.g., poly(ethylene glycol) diacrylate (PEGDA), poly(ethylene glycol) dimethacrylate (PEGDMA), poly(ethylene glycol) divinyl sulfones, and poly(ethylene glycol) diacrylamide) [5,6,7,8,9]. 

Two typical methods are applied to load a drug into a hydrogel. First, the reactive components can be initially mixed with the drug and form a crosslinked drug-containing hydrogel after a curing treatment. Second, a crosslinked hydrogel can be swollen in a solution of the drug until an equilibrium state is achieved [10]. The release of the loaded drug from the hydrogel matrix depends on its diffusion due to concentration gradients, matrix degradation, and swelling [11]. In addition, the material properties of the hydrogel, its surface-area-to-volume ratio, and the physical and chemical interactions between the drug and hydrogel significantly influence the drug-release mechanism. As a representative drug-mimetic substance, bovine serum albumin (BSA) can be used as a model protein drug. Rhodamine B (Rh-B) is also a good model because it can be stably retained in the network due to steric hindrance and is structurally similar to many hydrophilic analgesics and anti-inflammatory drugs [12,13,14,15].

Lin et al. [16] reviewed the different polymerization modes of PEG-based hydrogels and their physical properties for regenerative medicine applications. In general, a denser hydrogel network delivered improved mechanical properties, such as modulus and stiffness, but the permeability, diffusivity, water content, gel swelling, and hydrolytic degradation rate were lower. It is important to properly adjust the crosslinked network in the hydrogel according to the target application. Bryant et al. [17] regulated the network structure by inserting the synthesized PEGDMA of different proportions into the curable mixture. Weber et al. [18] aimed to elaborately synthesize PEGDMA of various molecular weights by reacting linear PEG with methacrylic anhydride to evaluate protein diffusion performance within the resulting hydrogel networks. In addition, Browning et al. [19] enhanced the crosslinked network by adding a four-arm PEG acrylate crosslinker to linear PEGDA hydrogels of different molecular weights. Although it is well known that the degree of crosslinking can be altered by the formulation of a hydrogel, few studies have attempted to control the hydrogel network through changes in the crosslinking process [20].

For PEG-based hydrogels specifically, Hwang et al. [21] systematically investigated real-time crosslinking behavior for hydrogel mixtures with different photo-initiator (PI)-to-crosslinker ratios, surface mechanical properties, and network configuration by varying the swelling ratio and gel fraction. Jung et al. [22] additionally considered the effect of the solvent (in this case, water and ethanol) on the crosslinking features and loading/release patterns of Rh-B in PEG hydrogels. Similarly, O’Donnell et al. [23] evaluated the permeability and release across the PEGDA hydrogel films using an Rh-B solution.

In this study, the evolution of crosslinked networks of PEG hydrogels according to the crosslinking method, i.e., UV or thermal curing, was investigated. One PEGMA monomer and two PEGDMA crosslinkers with different molecular weights were combined to produce hydrogels with different network structures and characteristics. To realize radical polymerization by either the UV or thermal curing method, 2,2-dimethoxy-2-phenylacetophenone (DMPA) and cyclopentane-*N*,*N*′-dicyclohexylcarbo diimide (C-PenDCC) were added as the photo-initiator (PI) or thermal radical initiator (TRI), respectively. Process conditions, such as the curing temperature in the thermal curing case and irradiation time in the UV curing case, were changed. During the curing processes, the relative degree of crosslinking was predicted using real-time Fourier-transform infrared (FT-IR) spectrometry, and real-time rheological properties were measured using a rotational rheometer in the small amplitude oscillatory shear (SAOS) mode. The swelling ratio, gel fraction, and loading and release of Rh-B were also compared for the cured hydrogel films produced through different curing processes. In addition, the surface mechanical properties for the cured films were determined using a nanoscratch tester (NST).

## 2. Experimental

### 2.1. Materials

**Figure 1:**
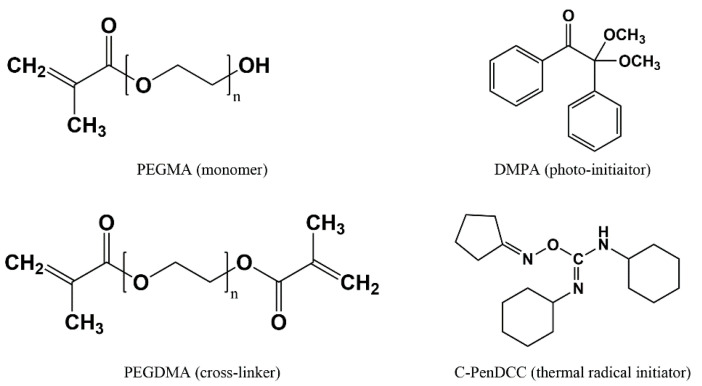
Chemical structures of poly(ethylene glycol) methacrylate (PEGMA), poly(ethylene glycol) dimethacrylate (PEGDMA), 2,2-dimethoxy-2-phenylacetophenone (DMPA), and cyclopentane-*N*,*N*′-dicyclohexylcarbo diimide (C-PenDCC).

The PEGMA (*M_w_* = 360 g/mol) monomer and two PEGDMA (*M_w_* = 330 and 750 g/mol) crosslinkers were purchased from Sigma-Aldrich (St. Louis, MO, USA) and Tokyo Chemical Industry (Tokyo, Japan). Note that the two PEGMDAs were used to ascertain the effect of the molecular weight on the hydrogel properties. DMPA, as the PI, was obtained from Sigma-Aldrich (St. Louis, MO, USA), and C-PenDCC, as the TRI, was supplied by the Korea Research Institute of Chemical Technology (KRICT, Ulsan, Korea) (Figure 1). C-PenDCC is an *O*-imino-isourea-based TRI derivative with a cyclopentane group that was used to adjust the dissociation energy and polymerization efficiency; using differential scanning calorimetry, it was found to have onset and peak curing temperatures of 65 and 74 °C, respectively [24]. The molar ratio of the monomer to the crosslinker was fixed at 1:1 to only focus on the effect of the PEGDMA’s molecular weight. The amount of initiator was 1.0 wt% based on the total amount of the monomer and crosslinker. Various UV- and thermally-curable PEG hydrogel mixtures with different formulations were prepared, as listed in Table 1.

### 2.2. Analysis of Real-Time Crosslinking Behavior

#### 2.2.1. Real-Time FT-IR

The chemical reactions to prepare the hydrogel were monitored in real-time during the UV and thermal curing stages with an attenuated total reflectance (ATR)-FT-IR spectrometer (Cary 600, Agilent Technologies, Santa Clara, CA, USA) equipped with UV and heating modules. Full FT-IR spectra were obtained during each reaction. In the UV curing case, the UV intensity was set to 1.32 mW/cm^2^ and the irradiation times were varied from 0 to 200 s at room temperature (RT, 25 °C). The reactions of the C=C double bonds in the methacrylate groups of PEGMA and PEGDMA were evaluated directly using the peak area of their IR band at 1637 cm^−1^. The conversion of the C=C bonds was evaluated using transient changes in the absorbance peak areas at 1637 cm^−1^ before and after curing. In the thermal curing case, the reactivity of the C=C double bonds was examined under several constant temperature conditions for 50 min.

Additionally, to probe the combined effects of the UV and thermal curing processes, the UV intensity and temperature were set to 1.32 mW/cm^2^ and 90 °C, respectively, and the UV irradiation time was changed from 0 to 150 s.

#### 2.2.2. Real-Time Rheological Properties

The real-time rheological properties (i.e., the storage modulus) of the UV or thermally curable hydrogel mixtures were measured using a rotational rheometer with a convection heating chamber (MCR 301, Anton Paar, Graz, Austria) in the SAOS mode at 0.1% strain and with a 1 Hz frequency. A disposable 8 mm parallel plate was used and the gap between the upper and lower plates was maintained at 500 μm. In the UV curing case, the UV intensity was set at 1.32 mW/cm^2^ and the irradiation time was manually adjusted in the range of 0–200 s to apply different UV dose conditions, as in the FT-IR tests, at room temperature and 90 °C. In the thermal curing case, the selected temperatures were constantly applied (70, 80, 90, 100, and 110 °C) for 30–50 min.

### 2.3. Measurements of Cured Hydrogel Film Properties

#### 2.3.1. Gel Fraction and Swelling Ratio

The changes in the gel fraction and swelling ratio of the several cured hydrogel films, which were measured via the rheological tests, were checked after the curing processes. The films were first dried in a vacuum oven for 24 h at 80 °C. The dried samples were weighed and then soaked in deionized (DI) water at room temperature for 72 h to extract the unreacted parts inside them. Then, the swollen films were removed from the DI water and reweighed after the wetted surfaces were carefully wiped. The swollen films were again completely dried in a vacuum oven for 24 h at 80 °C. The water swelling ratio (*q* = *W_s_*/*W_d_*) was defined as the ratio of the weight (*W_s_*) of the swollen film after the swelling test to the weight (*W_d_*) of the initial dried film before swelling. The gel fraction was calculated using the ratio (*f*(%) = *W_ex_*/*W_d_* × 100) of the completely dried weight (*W_ex_*) of the hydrogel film after the extraction of water-soluble constituents to the weight (*W_d_*) of the film before extraction [21].

#### 2.3.2. Rh-B Loading and Release

A stock solution of Rh-B (0.05 mM) was prepared and its absorbance was measured at a wavelength of 554 nm using a UV-visible spectrophotometer (Agilent 8453, Agilent Technologies, USA). Rh-B was incorporated into the several cured hydrogel films by soaking them in the Rh-B stock solution. After 48 h, the Rh-B-loaded hydrogel films were removed from the stock solution and their absorbance was measured to qualitatively predict the loading amount of Rh-B. The Rh-B-loaded films were placed in DI water to release the Rh-B from the hydrogel films. After 48 h, the hydrogel films were removed from the DI water and their absorbance was remeasured. The amounts of loaded and released Rh-B were calculated using the absorbance data [22].

#### 2.3.3. Penetration Scratch Resistance via the Nanoscratch Test

A nanoscratch tester (NST^3^, Anton Paar Tritec SA, Corcelles, Switzerland) was employed to compare the surface mechanical properties of the cured hydrogel films [25,26]. A spheroconical-type indenter was used to apply a gradual normal force when moving in the horizontal direction on the film’s surface with a scratching speed of 2 mm/min over a distance of 1 mm. The surface resistance of the cured films as a function of the normal force could be estimated by the penetration depth profile according to standard ASTM D 7187 [27]. In this test, a progressive mode was adopted in which the normal force was increased linearly in the range of 0.1 to 40 mN.

## 3. Results and Discussion

### 3.1. Real-Time Crosslinking Dynamics of Hydrogels during UV Curing

**Figure 2:**
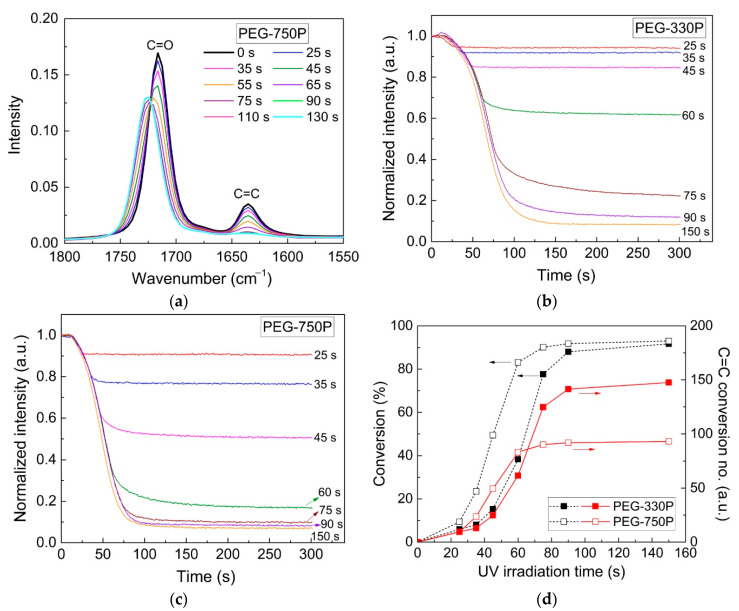
FT-IR intensity data for (**a**) a PEG-750P mixture and transient intensity changes of (**b**) PEG-330P and (**c**) PEG-750P mixtures during UV curing under different irradiation time conditions. (**d**) Changes in the conversion value and the actual number of C=C bonds participating in the reaction for the two cases.

The real-time crosslinking behaviors of hydrogel mixtures prepared with the PEGMA monomer and two PEGDMA crosslinkers, as listed in Table 1, were analyzed using FT-IR spectrometry as a function of the UV irradiation time. The temporal changes in the peaks of C=C (1637 cm^−1^) and C=O bonds (1715 cm^−1^) in the methacrylate group were confirmed via FT-IR spectral analysis. Figure 2a displays the representative real-time FT-IR spectra for a PEG-750P mixture under different UV irradiation times. The C=C peak intensity gradually decreased with increasing UV irradiation time, resulting in the formation of more densely crosslinked networks through more extensive radical polymerization. Note that the C=O peak in the methacrylate group was slightly shifted toward a higher wavenumber, from 1715 to 1724 cm^−1^, implying an increased carbonyl bond strength due to the loss of conjugation between the olefin and carbonyl groups during UV curing, as is already substantiated in the literature [28,29].

Figure 2b for PEG-330P and Figure 2c for PEG-750P show the real-time changes in the C=C peak intensity normalized to the initial values for various UV irradiation times. In both cases, radical polymerization by the PI (observed by the reduction of C=C peak intensity) was clearly and dominantly activated during the UV irradiation. Conversions of the C=C bonds in Figure 2d were evaluated using the change in C=C peak intensity before and after UV curing (at 300 s). For the same UV irradiation time, the conversion of PEG-750P (with the high *M_w_* PEGDMA) was higher relative to that of PEG-330P (with the low *M_w_* PEGDMA). This was because the total number of C=C bonds per unit volume of the PEG-330P system was 1.6 times larger than that in the PEG-750P system. For this reason, the portion of C=C bonds engaged in the radical reaction in PEG-750P was higher than that in PEG-330P at an early stage of the UV irradiation, such that the network was formed faster in PEG-750P than in PEG-330P in the initial and developing stages. As the UV irradiation time further increased, the difference in the conversions between the two hydrogel mixtures gradually decreased. Note that Figure 2d also displays the changes in the actual number of C=C bonds participating in the radical reaction for the two hydrogel mixtures, based on the conversion values and the total number of C=C bonds. The final hydrogel properties in the crosslinked networks were closely related to the number of C=C bonds participating in the polymerization. It can be shown that a relatively larger number of C=C bonds in the PEG-330P were involved in the reaction after the developing stage beyond the corresponding number of C=C bonds in the PEG-750P, unlike the trend in conversion changes for the two cases.

**Figure 3:**
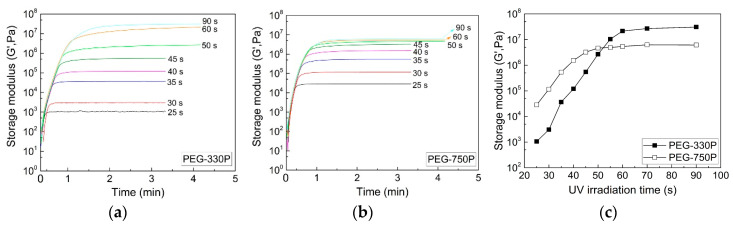
Real-time storage moduli for (**a**) PEG-330P and (**b**) PEG-750P mixtures during UV curing under different irradiation time conditions. (**c**) Maximum G′ data for the two hydrogel mixtures.

The real-time crosslinking behavior for the hydrogel systems could be also evaluated in terms of the evolution of their rheological properties (i.e., storage modulus) using a rotational rheometer in the SAOS mode, as displayed in Figure 3a for PEG-330P and Figure 3b for PEG-750P. As in the FT-IR spectra, the storage modulus (G′) increased as more UV irradiation energy (i.e., irradiation time) was applied to the hydrogel mixtures due to enhancement of the radical polymerization rate through the generation of more radicals (Figure 3c). Early in the reaction, the G′ of PEG-750P was higher than that of PEG-330P. However, as the UV irradiation time gradually elapsed, the G′ of PEG-330P grew rapidly and progressed beyond that of PEG-750P after about 52 s of irradiation (for a UV irradiation energy of ≈70 mL/cm^2^). This can be reasonably understood by the changes in the number of C=C bonds and the crosslinked network in both systems, as described in Figure 2d, which both qualitatively exhibit the same tendencies, albeit with slight differences in the crossover points.

### 3.2. Real-Time Crosslinking Behaviors of Hydrogels during Thermal Curing

**Figure 4:**
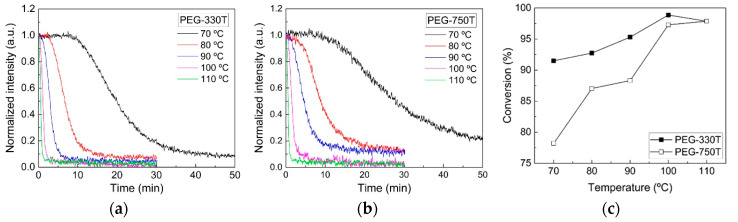
Transient intensity changes of (**a**) PEG-330T and (**b**) PEG-750T mixtures during thermal curing under different temperature conditions. (**c**) Changes in the conversion value for the two hydrogel mixtures.

Figure 4a,b presents the real-time crosslinking behaviors of the PEG-330T and PEG-750T hydrogel mixtures, respectively, under different thermal curing conditions in terms of the normalized FT-IR spectral peak intensities of the C=C bonds. Consistent with the role of the TRI, the C=C peak intensities rapidly decreased with increasing curing temperature. At relatively low temperatures (e.g., 70 and 80 °C), the reaction was delayed for several minutes because more energy was needed to activate the TRI. Thermal curing has the advantage of crosslinking curable materials in complex structures and light-inaccessible areas, although the reaction is slow in contrast to the photo-curing reaction.

Figure 4c shows the conversions of the two hydrogel mixtures based on changes in the C=C peak intensities between the initial and final states in the thermal curing (30–50 min). At the same temperature, the conversion of PEG-330T via thermal curing is higher relative to that of PEG-750T. In contrast to the UV curing process, with its sufficient and high energy for the radical reaction and the fast generation of radicals due to the PI, radical formation by the TRI might be a rate-determining step in the thermal curing reaction. In this case, it is thought that the relatively larger number of C=C bonds in PEG-330T than in PEG-750T is more advantageous for creating the crosslinked network during the rather slow thermal curing process. As the curing temperature rises, the conversion level for PEG-330T and PEG-750T would be similar at an energy sufficient to activate TRI. Interestingly, in the PEG-330T reaction, the conversion at 100 °C reached a maximum among the temperatures considered in this study, presumably resulting from the compromise between the fast reactivity and steric hindrance of the denser network structure at high temperatures.

**Figure 5:**
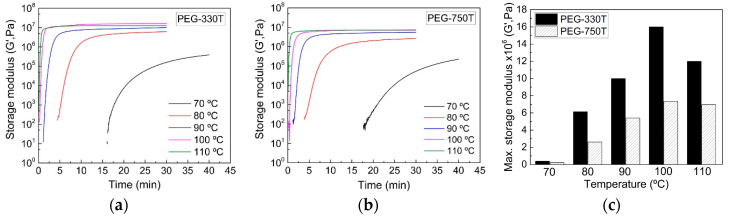
Real-time storage moduli for (**a**) PEG-330T and (**b**) PEG-750T mixtures during thermal curing under different temperature conditions. (**c**) Maximum G′ data for the two hydrogel mixtures.

Figure 5a,b depicts the real-time evolution of G′ for the PEG-330T and PEG-750T hydrogel mixtures under various curing temperature conditions. As in the UV curing process, G′ increased as the reaction temperature rose for both cases. The growth rate of G′ for PEG-330T was faster than for PEG-750T. Furthermore, the final G′ in PEG-330T was nearly 2 times higher than that of PEG-750T at the given temperatures (Figure 5c) because both the converted and number of C=C bonds participating in the radical reaction in the former were much higher than in the latter. In addition, G′ at 100 °C was higher than at 110 °C for PEG-330T, presumably indicating a denser crosslinked network at 100 °C, as described in the FT-IR analysis discussion. 

### 3.3. Temperature Effect on the UV Curing Process of the Hydrogels

**Figure 6:**
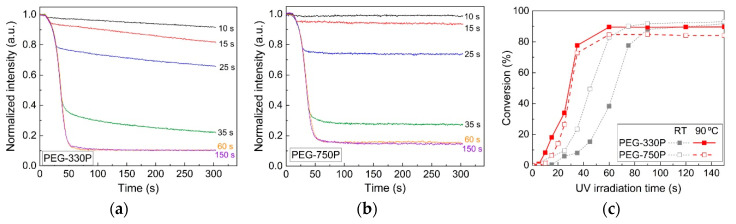
Transient intensity changes of (**a**) PEG-330P and (**b**) PEG-750P mixtures during UV curing under different irradiation times at 90 °C. (**c**) Changes in conversion value for the two hydrogel mixtures.

To examine the effect of temperature on the UV curing process, real-time FT-IR results (showing the temporal change of normalized C=C peak intensities) for hydrogel mixtures containing the PI (not the TRI) are presented in Figure 6a for PEG-330P and Figure 6b for PEG-750P at 90 °C. The intensities of the C=C peak greatly decreased with increasing UV irradiation time in the high-temperature environment. Note that, after the specified period of UV irradiation, the continuous progress of the curing reaction in PEG-330P was evident. Figure 6c compares the conversions at 300 s after the UV irradiation of both PEG-330P and PEG-750P at room temperature (RT) or 90 °C. Note that the conversion results at RT in Figure 2d were replotted here. The conversion in PEG-330P was slightly higher than that of PEG-750P at 90 °C, as opposed that at room temperature (also see Figure 2d). The conversion at high temperature seemed to be affected by the higher diffusion of reactive molecules containing a large number of C=C bonds in PEG-330P. Note that the relatively low final conversion values at 90 °C in comparison with those at RT might be ascribed to steric hindrance by the initially fast-grown crosslinked network.

### 3.4. Swelling Ratio, Gel Fraction, and Rh-B Loading/Release Properties

**Figure 7:**
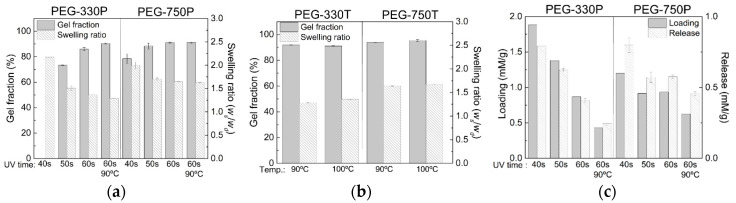
(**a**,**b**) Gel fraction and swelling ratio data, and (**c**) Rh-B loading and release amounts of PEG-300 and PEG-700 hydrogel films produced under the selected crosslinking conditions.

The gel fractions and swelling ratios of several hydrogel films of the PEG-330 and PEG-750 samples fabricated under selected crosslinking conditions were measured to qualitatively understand their network states, which were dependent on the molecular weight of the crosslinker and UV or thermal processing conditions (Figure 7a,b). In the UV curing process, the gel fraction increased with increasing UV irradiation time and its value was generally higher for PEG-750P than PEG-330P (Figure 7a), although there was little difference after the completion of the reaction. This was consistent with the conversion results observed in the FT-IR experiments. In contrast, the swelling ratio decreased as the UV irradiation time increased, representing the relatively greater extent of crosslinking with increasing UV irradiation time. The swelling ratio in PEG-750P was higher than in PEG-330P, which makes sense given that the latter forms more densely crosslinked networks at more crosslinking sites. It is worth mentioning that the degree of crosslinking of hydrogels can be roughly identified by the mesh size (*ξ*) from two crosslinking points (*M_c_*) using Equations (1) and (2) [21,30]:

(1) $$M_{C}=\frac{n\left(M\right)}{n\left(C\right)}M\left(M\right)+M\left(C\right),$$

(2) $$\xi =l\left(\frac{2M_{c}}{\bar{M}}\right)C_{N}^{\frac{1}{2}}q^{\frac{1}{3}},$$

where *M*(M) and *M*(C) are the molecular weights of the monomer and crosslinker, respectively, and *n*(M) and *n*(C) are the number of moles of the monomer and crosslinker, respectively. *l* is the length of a C–C single bond (*l* = 0.154 nm), $\bar{M}$ is the average molecular weight of the monomer and crosslinker, *C_N_* is the characteristic ratio (6.9 for acrylates) [31], and *q* is the swelling ratio. For instance, the mesh size of hydrogels in Figure 7a decreased with increasing UV duration time (i.e., 2.096 (40 s), 1.855 (50 s), and 1.795 (60 s) for PEG-330P and 2.037 (40 s), 1.931 (50 s), and 1.911 (60 s) for PEG-750P) and decreasing crosslinker *M_w_*. 

Notably, the thermal curing process also exhibited the same trends as the UV curing process (Figure 7b). For example, the swelling ratio of PEG-330T, with its more densely crosslinked network and higher G′, was lower than that of PEG-750T.

The loading and release of Rh-B were measured as an indicator of the drug-loading capacity using several hydrogel films. Focusing on the UV-cured hydrogels (Figure 7c) in particular, both loading and release decreased with increasing UV irradiation time, confirming the same trend observed in the swelling ratio data. Note that the loading and release amounts rapidly decreased with UV irradiation time in PEG-330P compared to PEG-750P. This was because the denser network structure of the PEG-330P hydrogel hindered the ability of Rh-B to penetrate the crosslinked network to a greater extent. The difference between the Rh-B loading and release amounts was larger in PEG-330P relative to PEG-750P due to the different interactions between the hydrogel networks and the Rh-B, as explained by Das et al. [32].

### 3.5. Surface Mechanical Properties of Hydrogel Films

**Figure 8:**
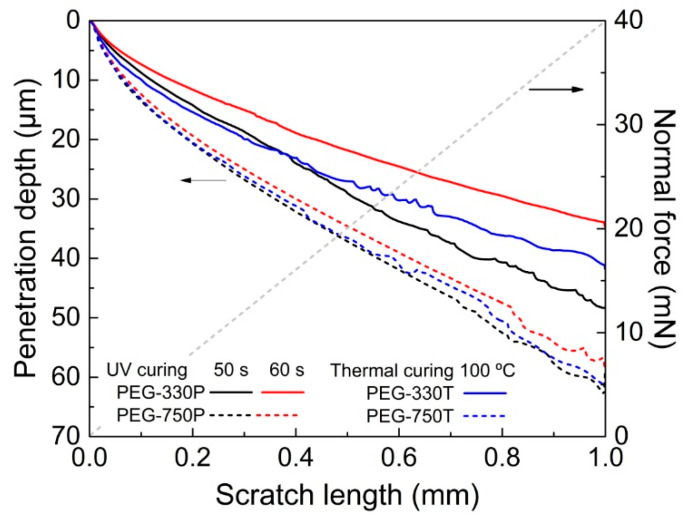
Penetration depths for several PEG-330 and PEG-750 hydrogels films produced via a UV or thermal curing process.

The penetration depths of several PEG hydrogel films selected from Figure 7 as a function of an applied normal force were compared using the NST to quantify the actual surface resistance of the films [21,22,24,26]. Figure 8 shows the penetration depth profiles for a gradual increase of the vertical load from 0 to 40 mN along a 1.0 mm distance on the film surface. The penetration depths were proportional to the molecular weight of the crosslinker. In other words, the PEG-330 films produced from the UV and thermal curing processes displayed shallower penetration depths than the PEG-750 films. This result was consistent with the evolution of G′ for the cured hydrogel films described above. Thus, the real-time crosslinking dynamics played a decisive role in properly tuning the hydrogel properties, depending on the conditions of the curing method.

## 4. Conclusions

The effects of the molecular weight of the crosslinker and the curing method (UV or thermal polymerization) on the crosslinking dynamics and gelation features of PEG-based hydrogels were investigated using one PEGMA monomer and two PEGDMA crosslinkers. From the FT-IR spectrometry and rotational rheometry, different crosslinking evolutions of PEG hydrogel mixtures were found, depending on the polymerization method and detailed conditions. Interestingly, in the UV curing, there was the crossover in developing both the conversion and storage modulus for PEG-300P and PEG-750P mixtures, which could be explained by the changes in the number of C=C bonds in the two systems. In the thermal curing, the conversion and G′ of PEG-330T were relatively higher than those of PEG-750T because the relatively large number of C=C bonds in the former facilitated the formation of crosslinked networks during the rather slow thermal curing. The gelation characteristics of UV- and thermally-crosslinked hydrogel films, including gel fraction, swelling ratio, Rh-B loading/release amount, and surface resistance from NST, correlated well with the real-time crosslinking behavior during each polymerization process. Thus, establishing a relationship between the crosslinking dynamics of hydrogel mixtures during the curing process and the final hydrogel properties will be important for their suitable application in various fields. 

## Figures and Tables

**Table 1:** Formulation of several pre-polymerized mixtures for UV and thermal polymerizations.

Polymers		PEG-330T (g)	PEG-750T (g)	PEG-330P (g)	PEG-750P (g)
PEGMA (Monomer)		0.522	0.324	0.522	0.324
PEGDMA * (Crosslinker)	*M_w_* = 330	0.478		0.478	
	*M_w_* = 750		0.676		0.676
C-PenDCC (TRI)		0.01	0.01	-	-
DMPA (PI)		-	-	0.01	0.01

* *M_w_* of PEGDMA in PEG-330 samples = 330 g/mol, *M_w_* of PEGDMA in PEG-750 samples = 750 g/mol.
